# Supplementary material for: Arctic freshwater outflow suppressed Nordic Seas overturning and oceanic heat transport during the Last Interglacial
Source: Nat Commun. 2024 Oct 27;15:8998. doi: 10.1038/s41467-024-53401-3 (PMC11514159; doi:10.1038/s41467-024-53401-3)
Supplement: Supplementary file 1 — Supplementary Information [file 41467_2024_53401_MOESM1_ESM.pdf]

Supplementary Information for:

Arctic freshwater outflow suppressed Nordic Seas overturning and oceanic heat transport during the Last Interglacial

Mohamed M. Ezat[1*], Kirsten Fahl[2], Tine L. Rasmussen[3]

[1] iC3 - Centre for ice, Cryosphere, Carbon and Climate, Department of Geosciences, UiT, The Arctic University of Norway, Norway.
[2] Alfred Wegener Institute Helmholtz Centre for Polar and Marine Research, Am Handelshafen 12, 27570 Bremerhaven, Germany.
[3] Department of Geosciences, UiT, The Arctic University of Norway, Norway.

* Correspondence to: M. M. Ezat (mohamed.ezat@uit.no).

This file includes:

Supplementary Figure 1

Supplementary Figure 2

Supplementary Figure 3

Supplementary Figure 4

Supplementary Table 1

Supplementary Figure 1. Bioturbation event in Core LINK16 (blue-highlighted). (a) Number of basaltic shards per gram counted in the >100 µm in core LINK16 (ref. 31). **(b)** Relative abundance of the benthic foraminiferal species *Cassidulina reniforme*. **(c)** Relative abundance of the polar planktic foraminiferal species *Neogloboquadrina pachyderma* in cores LINK16 (ref. 31; blue) and MD95-2009 (ref. 23; brown). The blue-highlighted area marks an interval in core LINK16 where is a large amplitude, short-term change in planktic and benthic foraminiferal assemblages at 124.5 ka – in particular an increase in the subpolar planktic foraminiferal species. These changes are not recorded in the other nearby Norwegian Sea cores JM11-FI-19PC and MD95-2009. At the same interval in LINK16, the concentration of basaltic shards decreased suggesting that this interval could have been affected by bioturbation from the red-highlighted interval. It is possible that a burrow has brought sediments that are rich in the subpolar planktic foraminifera and poor in basaltic grains from late LIG sediments (the red-highlighted interval). The bioturbation effects are clear in the basaltic shard counts, benthic and planktic fauna because of the high contrast in these parameters between the early and late LIG parts. The bioturbation effects are not clear in other proxies such as foraminiferal $\delta^{18}O$ and IP_{25} because these are not significantly different between the two intervals (Figs 1b,c, 2c). This highlights the importance of the multi-core investigation and needed caution with single core-based studies.

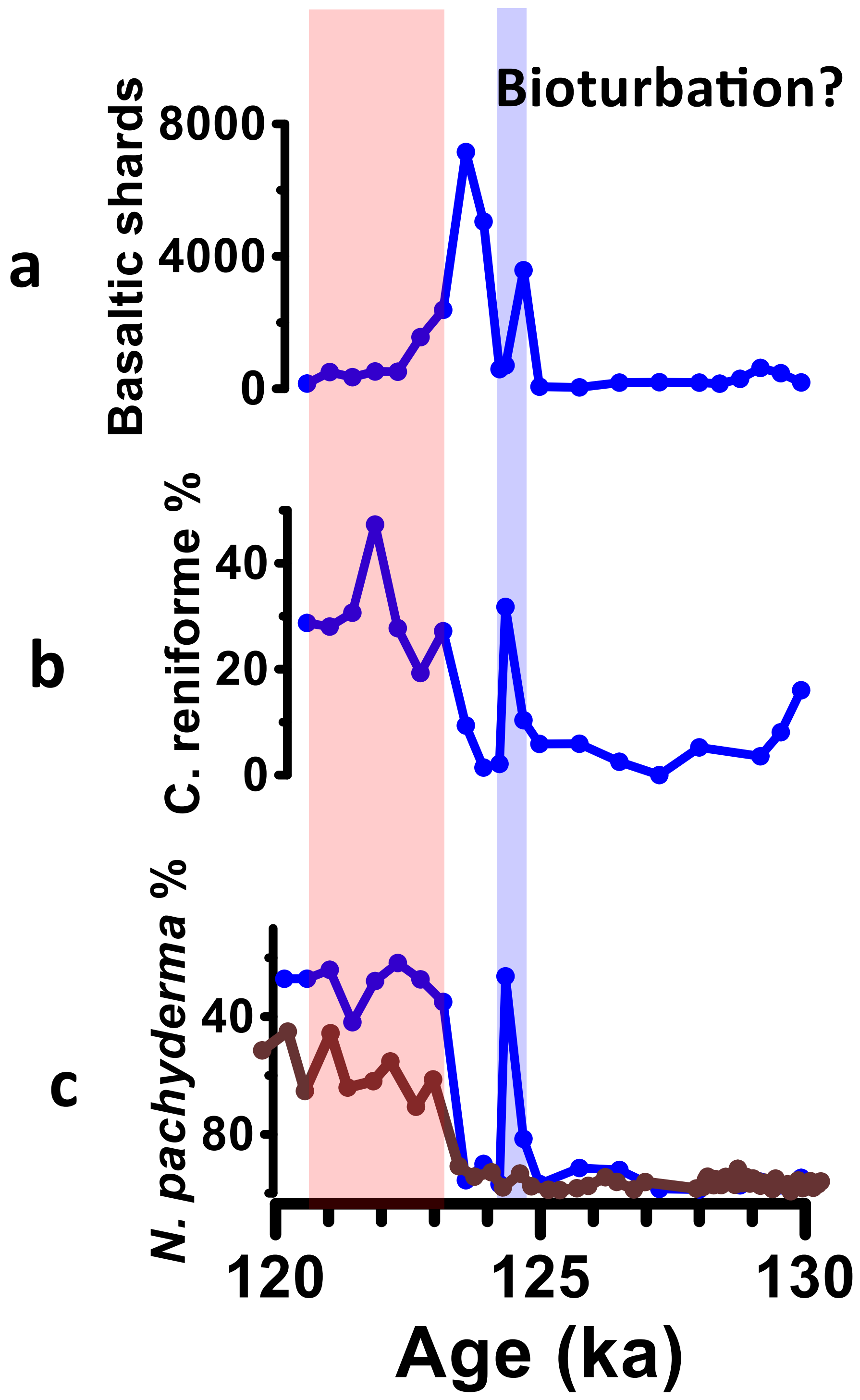
Bioturbation?
a
Basaltic shards
8000
4000
0
b
C. reniforme %
40
20
0
c
N. pachyderma %
40
80
120
125
130
Age (ka)

Supplementary Figure 2. (a) Relative abundance of sea-ice indicating diatom species[29]. **(b)** Relative abundance of the dinocyst sea-ice species *Islandinium minutum*[27]. **(c)** Sea ice index P_BIP_{25} from two sediment cores (this study). **(d)** Concentration brassicasterol from two sediment cores (this study). **(e)** Concentration of IP_{25} (a C_{25} Isoprenoid Lipid) from two sediment cores (this study). Temperature proxies. **(f)** Relative abundance of the polar planktic foraminiferal species *Neogloboquadrina pachyderma*[23,31]. Black, blue and brown colors refer to data from sediment core JM11-FI-19PC, LINK16 and MD95-2009, respectively (see Figure 1a for the core locations). MIS refers to Marine Isotope Stage.

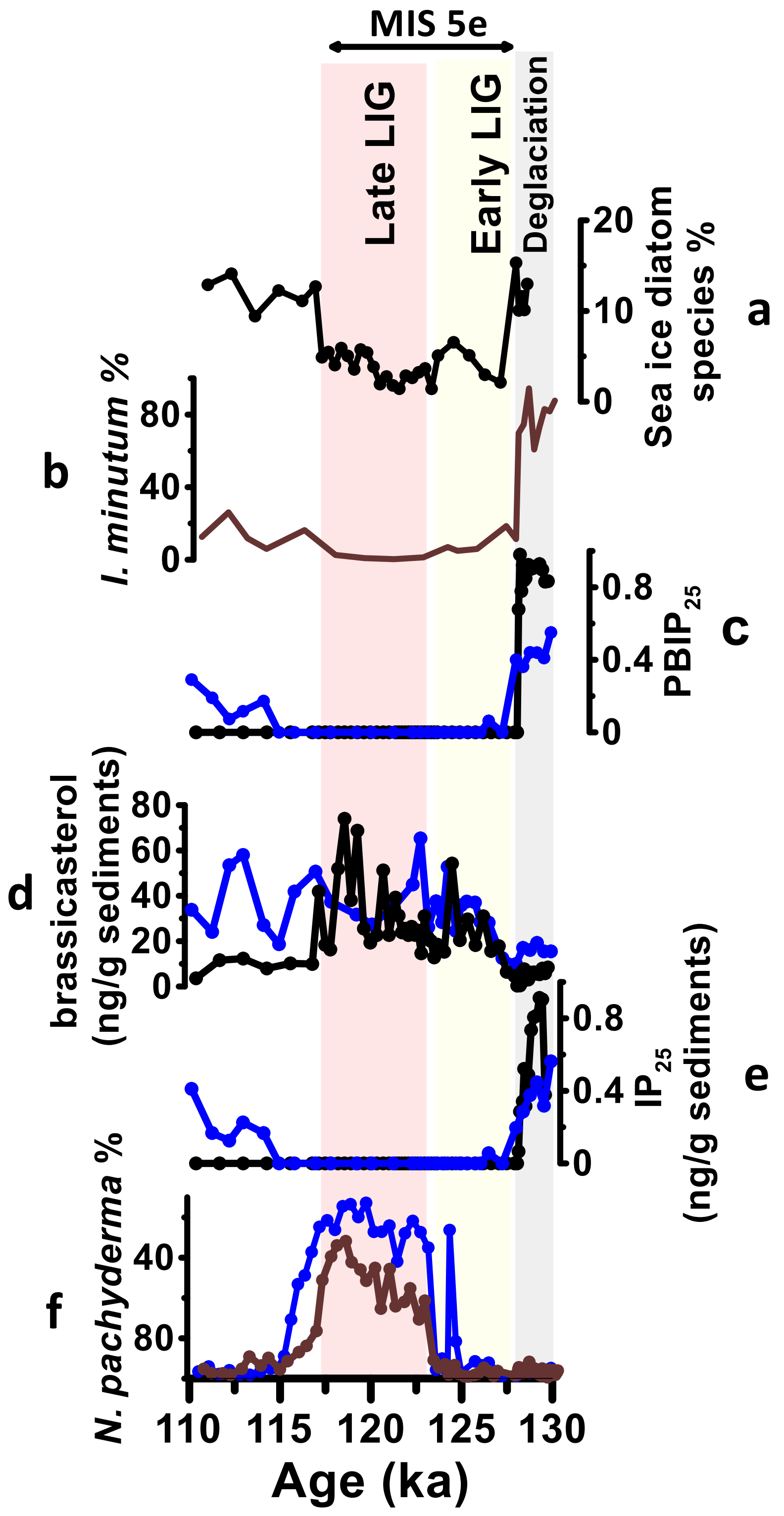

Supplementary Figure 3. $\delta^{13}C$ measured in the infaunal benthic foraminiferal species *M. barleanus* in sediment cores LINK16 (blue) and JM11-FI-19PC (black). The grey bar highlights the whole LIG interval. Infaunal benthic $\delta^{13}C$ responds to changes in the flux of organic matter and/or oxygen availability within their environment (the upper few centimetres in the sediments; e.g., ref. 71). Stable infaunal benthic $\delta^{13}C$ can be indicative on stable food supply to the seabed. It is noteworthy that epifaunal benthic $\delta^{13}C$ is often used as an indicator to changes in deep water mass sourcing and ventilation (e.g., ref. 72), but we could not find epifaunal benthic foraminifera in the early LIG interval.

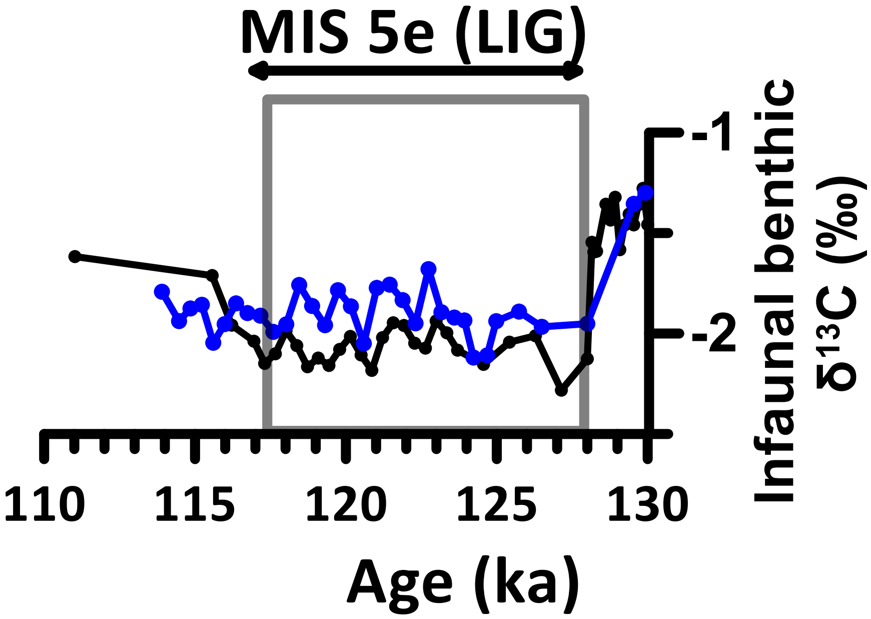

Supplementary Figure 4. Age models for sediment cores LINK16 and JM11-FI-19PC. (a) Percentage of *N. pachyderma* from core LINK16 (blue, ref. 31) and core MD95-2009 (brown, ref. 23). **(b)** Benthic foraminiferal $\delta^{18}O$ from cores JM11-FI-19PC (black, ref. 24), LINK16 (blue, this study), MD95-2009 (brown, ref. 27). **(c)** Planktic foraminiferal $\delta^{18}O$ from cores JM-FI-19PC (black, ref. 24), LINK16 (blue, this study), MD95-2009 (brown, ref. 23). The solid vertical lines refer to the main four tie points used in transferring the age model of MD95-2009 (ref. 26) to sediment cores LINK 16 and JM-FI-19PC. The dashed line refers to an additional age marker for the bottom of core JM-FI-19PC.

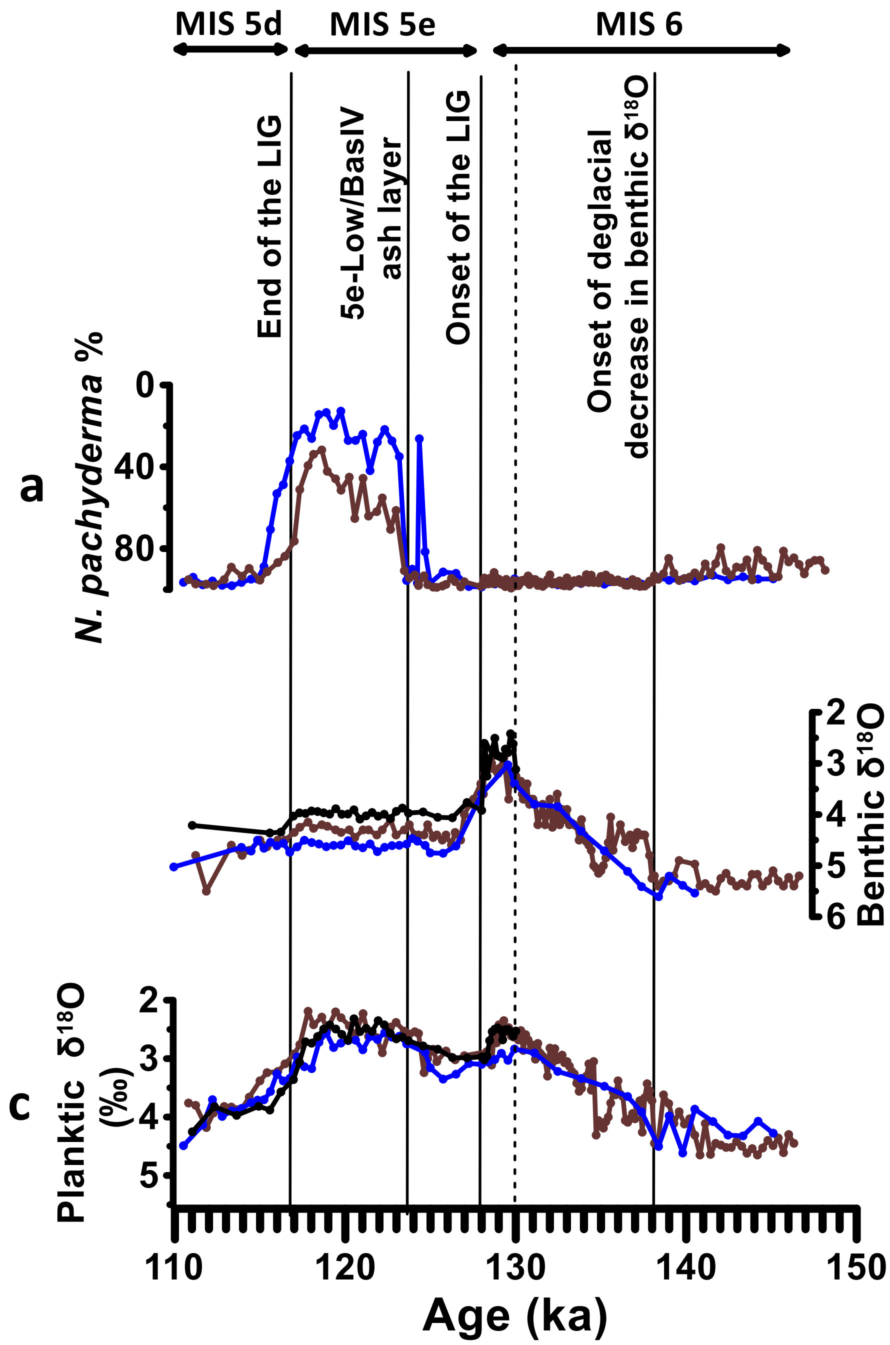

Supplementary Table 1. Tie points used in the age models of sediment cores LINK16 and JM-FI-19PC (see Supplementary Fig. 4).

**Tie point**	**Age (thousands of years before present, ka)**	**Depth (cm)**	
		JM-FI-19PC	***LINK16***
End of the Last Interglacial (LIG)	116.74	912	664
5e-Low/BasIV ash layer	123.7	1010	696.5
Onset of the LIG	128	1035	725
Additional tie point at the bottom of core JM-FI-19PC	130	1100	751
Onset of deglacial decrease in benthic $\delta^{18}O$	138.2		781

Supplementary References

71. Mackensen, A. & Schmiedl, G. Stable carbon isotopes in paleoceanography: atmosphere, oceans, and sediments. *Earth-Science Reviews* **197**, 102893 (2019).

72. Duplessy, J. C., Shackleton, N. J., Fairbanks, R. G., Labeyrie, L., Oppo, D. & Kallel, N. Deepwater source variations during the last climatic cycle and their impact on the global deepwater circulation. *Paleoceanography* **3**, 343-360 (1988).
