# Supplementary material for: Arctic freshwater outflow suppressed Nordic Seas overturning and oceanic heat transport during the Last Interglacial
Source: Nat Commun. 2024 Oct 27;15:8998. doi: 10.1038/s41467-024-53401-3 (PMC11514159; doi:10.1038/s41467-024-53401-3)
Supplement: Supplementary file 2 — Description of Additional Supplementary Files [file 41467_2024_53401_MOESM2_ESM.pdf]

Supplementary Dataset 1: Biomarker, foraminiferal abundance, stable isotope and trace element data from sediment cores JM11-FI-19PC and LINK16. Sheet **(1)** includes brassicasterol (24-methylcholesta-5, 22E-dien-3β-ol), IP25 (a C25 Isoprenoid Lipid), sea ice index P(B)IP25, benthic foraminiferal assemblages, and foraminiferal stable isotope data from sediment core LINK16. Sheet **(2)** contains brassicasterol, IP25 and planktic foraminiferal Na/Ca and Ba/Ca from sediment core JM11-FI-19PC. Sheet **(3)** includes the age model tie points of the two sediment cores. Abbreviations: ka, thousands of years before present; *N. pachyderma* d18O, oxygen isotope values measured in *Neogloboquadrina pachyderma*, unit permille (‰); *M. barleeanus* d13C, carbon isotope values measured in *Melonis barleeanus*; %*M. barleanus*, percentage of *Melonis barleeanus* of the total benthic foraminifera; %*C. neoteretis*: percentage of *Cassidulina neoteretis* of the total benthic foraminifera; and %*C. reniforme*: percentage of *Cassidulina reniforme* of the total benthic foraminifera.
